# Bibliometric Analysis of Mexican Publications on Stereotactic and Functional Neurosurgery From 1949 to 2021

**DOI:** 10.3389/fsurg.2022.886391

**Published:** 2022-05-09

**Authors:** José Damián Carrillo-Ruiz, Armando Armas-Salazar, José Luis Navarro-Olvera, Jesús Q. Beltrán, Brigham Bowles, Guillermo González-Garibay, Ángel Lee

**Affiliations:** ^1^Unit for Stereotactic and Functional Neurosurgery, and Research Direction, General Hospital of Mexico, Mexico City, Mexico; ^2^Faculty of Health Sciences Direction of Anahuac University Mexico, Mexico City, Mexico; ^3^Postgraduate Department, School of Higher Education in Medicine, National Polytechnic Institute, Mexico City, Mexico; ^4^Instituto Nacional de Neurología y Neurocirugía, Neuroendovascular Therapy, Mexico City, Mexico; ^5^Comisión Coordinadora de Institutos Nacionales de Salud y Hospitales de Alta Especialidad, Mexico City, Mexico

**Keywords:** bibliometrics, stereotactic surgery, functional neurosurgery, medical research, Mexico

## Abstract

**Background:**

Stereotactic and functional neurosurgery (SFN) is a rapidly evolving field and some emerging countries, especially Mexico, have made significant contributions to this discipline. A bibliometric analysis has never been performed in Latin America, and this would be particularly important to show the areas that remain poorly studied, and design research strategies for the future.

**Methods:**

Scopus was queried using keywords pertaining to functional neurosurgery, restricting the affiliation country to Mexico, and considering documents published after 1949. Added to the initial search, a complementary literature exploration by author, considering the publications of the most productive neurosurgeons, was performed. A descriptive statistical analysis was carried out.

**Results:**

From 5,109 articles, only 371 were eligible. Scientific production has gradually increased with time. Epilepsy (31%) and movement disorders (27.4%) were the most studied neurological conditions, whereas the other 41.6% corresponded to pain, behavior disorders, spinal cord injuries, neuromodulation, stereotactic biopsies, and SFN history. Level of evidence was predominantly level V (59.1%). Publication output is highly skewed to Mexico City, which represents 78.4% of national production. Relative to factors associated with impact of research, publications in English had more citations (28.5 mean citations per paper), and journals with an impact factor greater than one had more than 10 mean citations per paper.

**Conclusions:**

Mexico has experienced an increase in the productivity of SFN literature, addressing the most prevalent issues in the country (epilepsy and motor disorders). However, it is necessary to report studies with a higher level of evidence, as well as to decentralize the research collaborating with national institutions outside Mexico City. On the other hand, it is imperative to promote scientific production in English and in high-impact indexed journals to increase the visibility of our production. We would like to call upon our colleagues in other countries to reproduce our methodology, in order to determine the factors associated with the impact and productivity on SFN research.

## Introduction

The roots of stereotactic and functional neurosurgery (SFN) in Mexico date back to the 1930s, when non-neurosurgeons (Darío Fernandez, Mariano Vázquez) operated on patients for pain or epilepsy (1, 2). Academic Neurosurgery was initiated by Clemente Robles in the Hospital General de México (HGM, 1937). Later, Manuel Velasco-Suarez started at the Hospital Juárez (1944) and founded the Instituto Nacional de Neurología y Neurocirugía (INNN, 1964), the first institution in the country which focused exclusively on the treatment of neurological disorders (3–5). María Cristina Garcia-Sancho (first female neurosurgeon in Latin America) was also a pioneer in SFN (6, 7). To better understand the impact of these pioneers and other researchers and clinicians in this field, we intended to perform a bibliometric analysis. Such analyses can be regarded as the map of medical research and is a study tool using quantitative indicators to understand the effect of research productivity, describing the characteristics of the research and the factors associated with a greater impact (8–10). Previous papers on SFN focused on specific topics (neuromodulation, movement disorders, stereotactic biopsy, and spinal cord injuries) (11–14). There is only one article reported by Lipsman and Lozano (15) related to SFN that analyzes this discipline in a global manner (top-cited articles), considering the research output of different countries. However, productivity by country/institution was not mentioned by the authors. Moreover, SFN is a subspecialty, often requiring high-tech facilities, and research in resource-limited settings may shed additional light on topics not studied in wealthier countries. This study is the first analysis of the productivity of an entire nation. Recently, the first bibliometric analysis focusing on epilepsy in Mexico was published (8, 9, 16). The objectives of the present study were to know: (a) the most studied areas, (b) the factors associated with the impact of the publications, (c) the characteristics of the production, and (d) the most productive institutions and authors nationwide. This paper will also set the pace for a deeper analysis of the Latin American contributions in SFN.

## Methods

Database was harvested from Scopus by using specific keywords in the field: “Textual Content: All Fields,” restricting affiliation country to “Mexico,” while only considering documents published “after 1949.” The keywords included in the search strategy were the following: Balloon-compression, Brain-machine-interface, Callosotomy, Capsulotomy, Cingulotomy, Cordotomy, Deep-brain-stimulation, Dystonia-surgery, Dorsal-root-entry-zone, DREZ, Electrical-stimulation, Epidural-stimulation, Epilepsy-surgery, Hemifacial-spasms, Hemispherectomy, Image-guided-surgery, Intrathecal-infusion-pump, Language-mapping, Lobectomy, Lobotomy, Mesencephalotomy, Microvascular-decompression, Motor-cortex-stimulation, Movement-disorder-surgery, Neuronavigation, Neurotomies, Pallidotomy, Pain-surgery, Parkinson-surgery, Parkinson-transplant-surgery, Peripheral-nerve-stimulation, Prelemniscal-radiation-surgery, Psychosurgery, Psychiatric surgery, Radiofrequency, Rhizotomy, Spasticity-surgery, Spinal-cord-stimulation, Stereotactic, Stereotaxic, Stereotaxis, Subpial-transection, Subthalamotomy, Sympathectomy, Temporal-lobectomy, Thalamotomy, Tractotomy, Tremor-surgery, Trigeminal-neuralgia-surgery.

It was decided to use Scopus exclusively, not only because it is an appropriate source for citation tracking but also because many Mexican journals are not indexed in PubMed but are included in Scopus. The primary objective was to analyze the scientific production in the field of SFN. The inclusion criteria were: Mexican author, affiliated to a Mexican institution, any language, and related to topics concerning SFN, such as epilepsy, pain, movement disorders (including spasticity), spinal cord stimulation (spinal cord injuries), behavior (psychosurgery), stereotactic biopsies, neuromodulation, and translational research in the area of functional neurosurgery (experimental studies not with human subjects with the intention to transfer their conclusions to neurosurgical practice). Documents concerning radiosurgery, or basic science (not realized in humans without translational objective to neurosurgery) were excluded. Neuromodulation (deep brain stimulation [DBS] and transcranial magnetic stimulation [TMS]) is routinely indicated for all other categories. Even though neuromodulation therapies are usually used for all the previous categories (epilepsy, movement disorders, pain, and behavior), we deemed relevant to separate the category of neuromodulation because in our current practice lesional procedures are more common. For a comprehensive view on most bibliometric aspects, our recorded variables were: temporal profile, subject of research, language of publication, type of research, journal of publication, level of evidence, geographic distribution of research output, international collaborations, leading authors and institutions in Mexico, and number of citations (to determine the five most cited articles). Descriptive statistics consisted of percentages to describe subject frequency based on the number of papers. The impact of scientific output was evaluated by the ratio of citations to publications (citations per publication or c/p).

**Figure 1:**
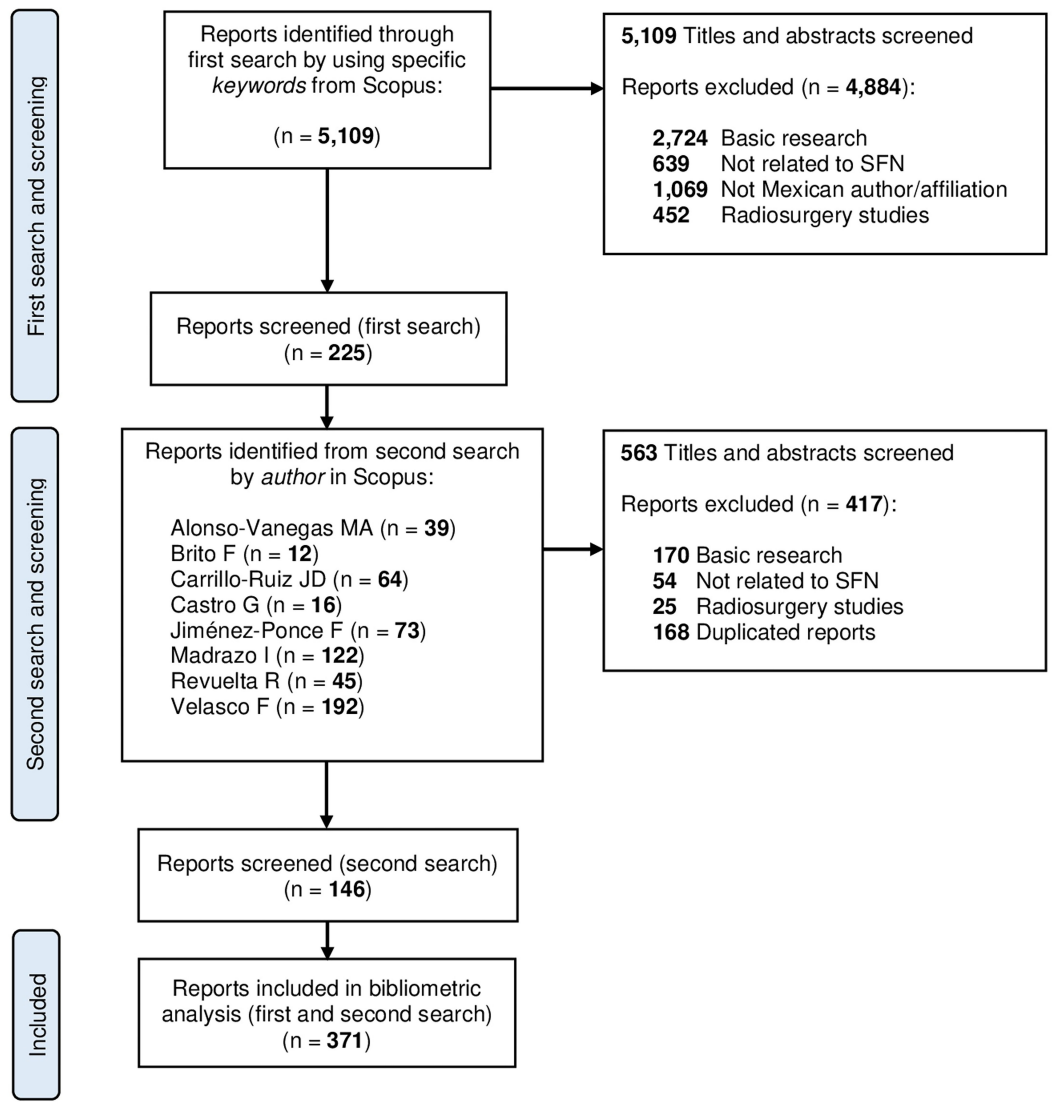
Flow diagram for search strategy.

The reports were manually selected (title and abstract) by a first reader and checked by a second evaluator. The study was intended to be carried out through a single search in Scopus using the previously mentioned keywords. However, the results of the first search significantly undercalculated the Mexican scientific productivity. For that reason, it was decided to complement the initial search by including the publications of the most productive neurosurgeons; these authors were defined as those who were recognized as neurosurgeons and had a number of publications ≥10 (Velasco F, Madrazo I, Jiménez F, Carrillo-Ruiz JD, Alonso Vanegas M, Revuelta R, Castro G, and Brito F) (shown in Figure 1). The two searches were carried out by the same evaluators, and the same inclusion and exclusion criteria were used for screening. Software was used for the development of bibliometric networks (VOSviewer version 1.6.16).

## Results

**Figure 2:**
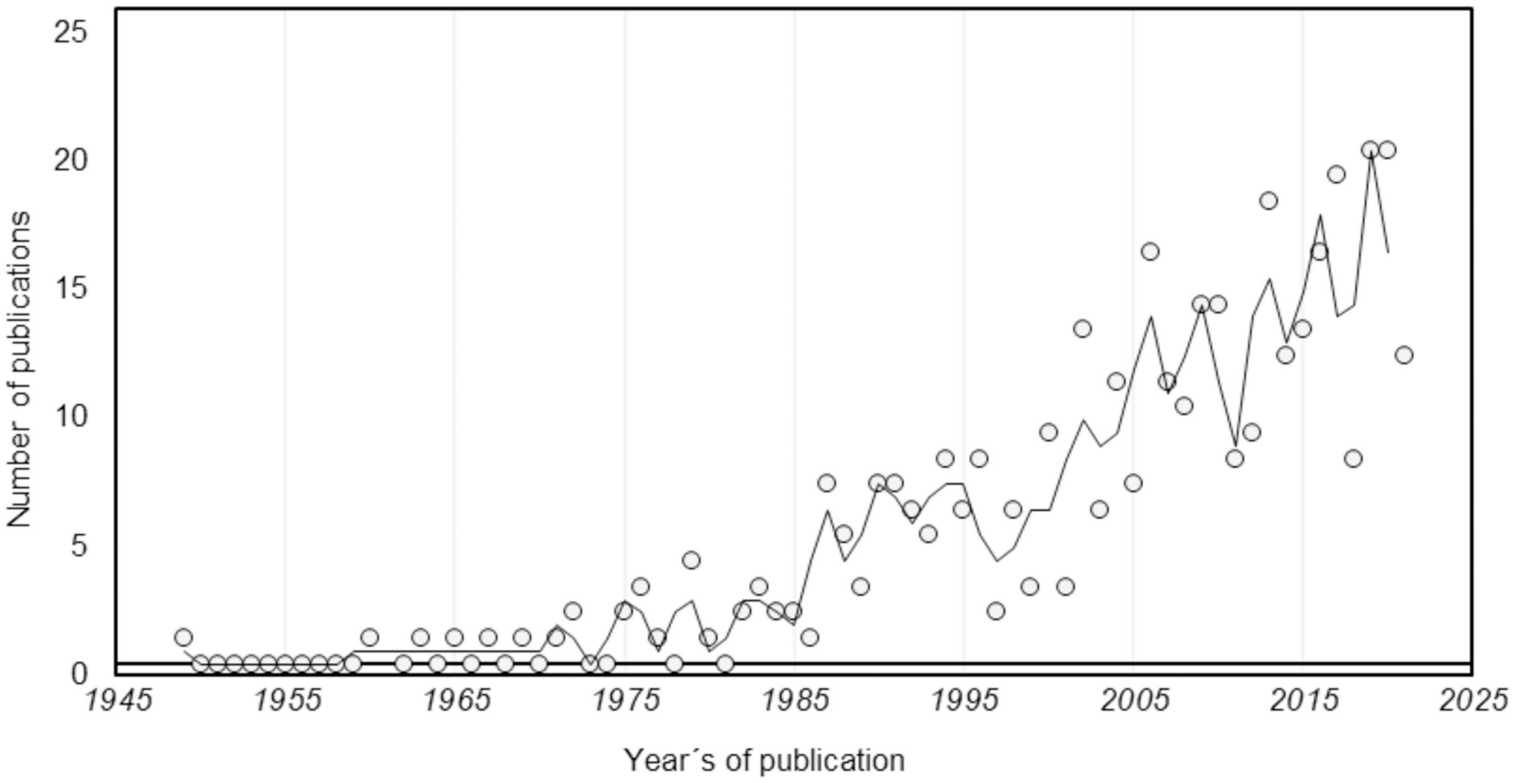
Chronological evolution of scientific production in Stereotactic and Functional Neurosurgery in Mexico.

**Figure 3:**
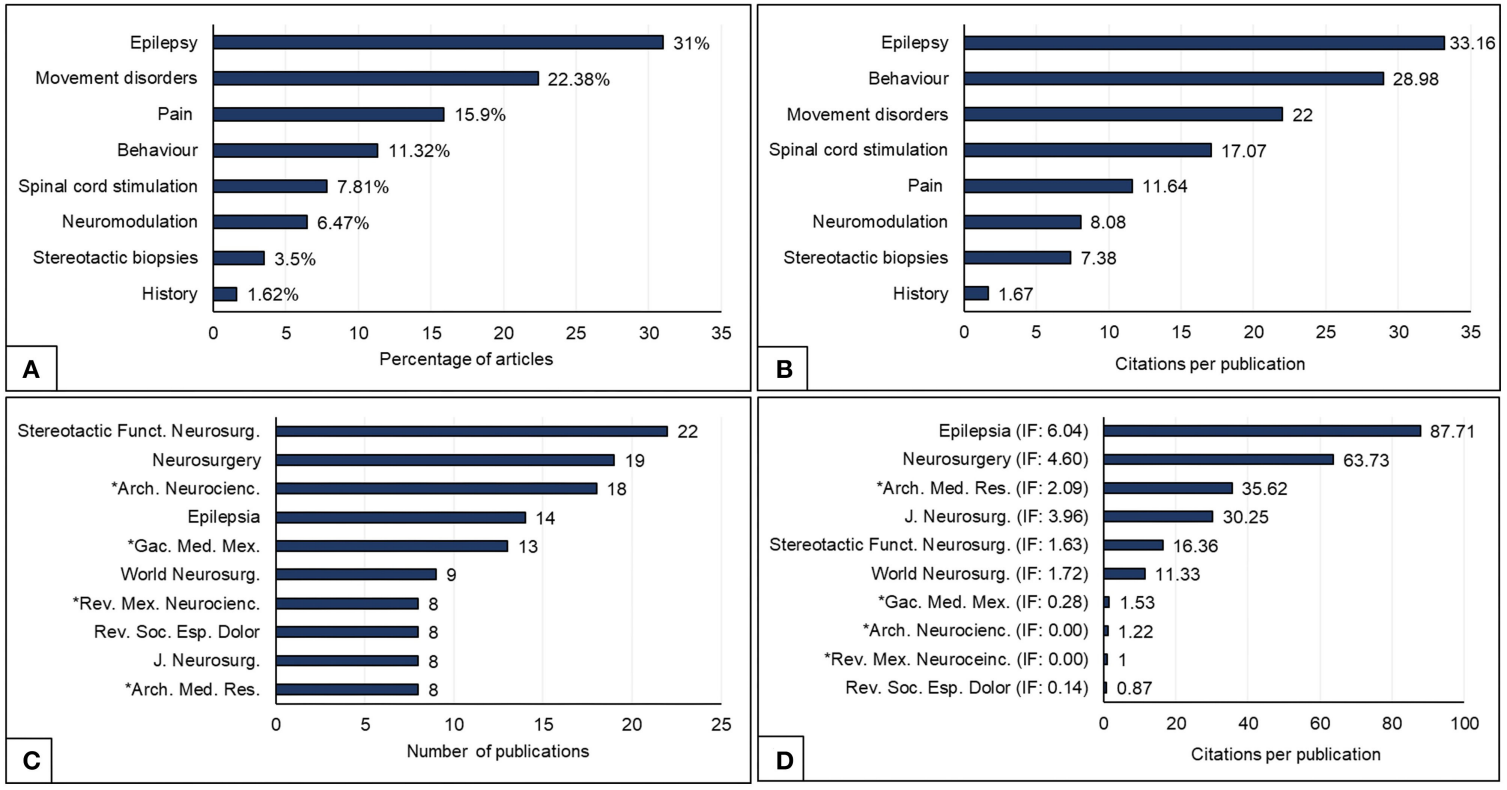
**(A)** Most frequent topic of publication. **(B)** Average number of citations per publication (divided by topic). **(C)** Most frequent journal of publication. **(D)** Number of citations per journal of publication. Asterisks represent the Mexican journals.

From 5,109 reports, 225 reports were obtained from the first search, and a total of 146 reports were added from the second search (by removing duplicate records and excluding those not dealing with SFN), thereby obtaining a total result of 371 reports that were included for the analysis from 1949 to 2021 (shown in Figure 1). A growing trend in scientific production of Mexico is shown in Figure 2. The main subject of study was epilepsy, which was 31% of the total production (shown in Figure 3A). The greater impact, citation per publication (c/p), was observed in epilepsy (33.2) (shown in Figure 3B). Most articles were published in English (77.4%), followed by Spanish (21.8%) and French (0.8%), with the greatest impact being for English (28.5), and much lower in the other two: French (2.7) and Spanish (1.9). Most articles were clinical (83.1%), compared to translational (15.9%). The types of documents were categorized as follows: articles (72.8%), reviews (14.2%), letters (5.1%), conference papers (3.2%), book chapters (3.2%), notes (1.9%), and short surveys (0.5%). According to the level of evidence, the documents about clinical research were predominantly case reports, case series, narrative reviews, and expert opinions (59%, level V evidence), followed by non-randomized or non-controlled experimental studies (29.6 %, level IV evidence), observational studies (10.6%, level III evidence), randomized and controlled trials (4.8%, level II evidence), and finally systematic reviews, meta-analysis, and clinical practice guidelines (1.6%, level I evidence). The preferred journals were: “Stereotactic and Functional Neurosurgery” (n = 22), “Neurosurgery” (*n* = 19), and “*Archivos de Neurociencias*” (*n* = 18) (shown in Figure 3C), is the national journal with the highest number of papers. When categorizing the journals according to their impact factor (IF), those with an IF > 1 had on average > 10 c/p, while those with an IF <1 had 1.5 c/p. The Mexican journal “Archives of Medical Research” (2019 IF = 2.1) displays an average of 35.6 c/p. The mean values of the c/p, and the medians (with minimum and maximum ranges), were the following for the most preferred journals: “*Epilepsia*” 87.7 (58.5: 1–215), “Neurosurgery” 63.7 (37: 0–349), “Archives of Medical Research” 35.6 (18: 5–122), “Journal of Neurosurgery” 30.2 (15.5: 0–90), “Stereotactic and Functional Neurosurgery” 16.3 (3: 0–86), “World Neurosurgery” 11.3 (1: 0–71), “*Gaceta Médica de México*” 1.5 (0: 0–7), “*Archivos de Neurociencias*” 1.2 (1: 0–4), “*Revista Mexicana de Neurociencia*” 1 (1: 0–2), and “*Revista de la Sociedad Española del Dolor*” 0.8 (1: 0–2) (shown in Figure 3D), thereby revealing that the Mexican journal with the greatest impact was “*Archives of Medical Research*.” The most cited articles are shown in Table 1, displaying the institutions with the greatest impact: Instituto Nacional de Ciencias Médicas y Nutrición Salvador Zubirán (INCMNSZ), Centro Médico Nacional “La Raza” (CMLR), and the HGM.

**Table 1:** Ten most cited articles.

**Institution**	**Article title**	**Year**	**Citations**	**Journal**
INCMNSZ	Long-term seizure outcomes following epilepsy surgery: A systematic review and meta-analysis.	2005	675	Brain
CMLR	Open Microsurgical Autograft of Adrenal Medulla to the Right Caudate Nucleus in Two Patients with Intractable Parkinson's Disease.	1987	534	NEJM
HGM	Electrical stimulation of the hippocampal epileptic foci for seizure control: A double-blind, long-term follow-up study.	2007	223	Epilepsia
HGM	Subacute electrical stimulation of the hippocampus blocks intractable temporal lobe seizures and paroxysmal EEG activities.	2000	215	Epilepsia
CMLR	Transplantation of fetal substantia nigra and adrenal medulla to the caudate nucleus in two patients with Parkinson's disease.	1988	202	NEJM
INCMNSZ	Long-term outcomes in epilepsy surgery: Antiepileptic drugs, mortality, cognitive and psychosocial aspects.	2007	201	Brain
HGM	A patient with a resistant major depression disorder treated with deep brain stimulation in the inferior thalamic peduncle.	2005	183	Neurosurgery
HGM	Electrical brain stimulation for epilepsy.	2014	180	Nat Rev Neurol. .
HGM	Electrical Stimulation of the Centromedian Thalamic Nucleus in the Treatment of Convulsive Seizures: A Preliminary Report.	1987	171	Epilepsia
HGM	Double-blind, randomized controlled pilot study of bilateral cerebellar stimulation for treatment of intractable motor seizures.	2005	153	Epilepsia

*INCMNSZ, Instituto Nacional de Ciencias Médicas y Nutrición “Salvador Zubirán”; CMLR, Centro Médico La Raza; HGM, Hospital General de México*.

### Relevant Institutions and Authors in Stereotactic and Functional Neurosurgery Production

**Figure 4:**
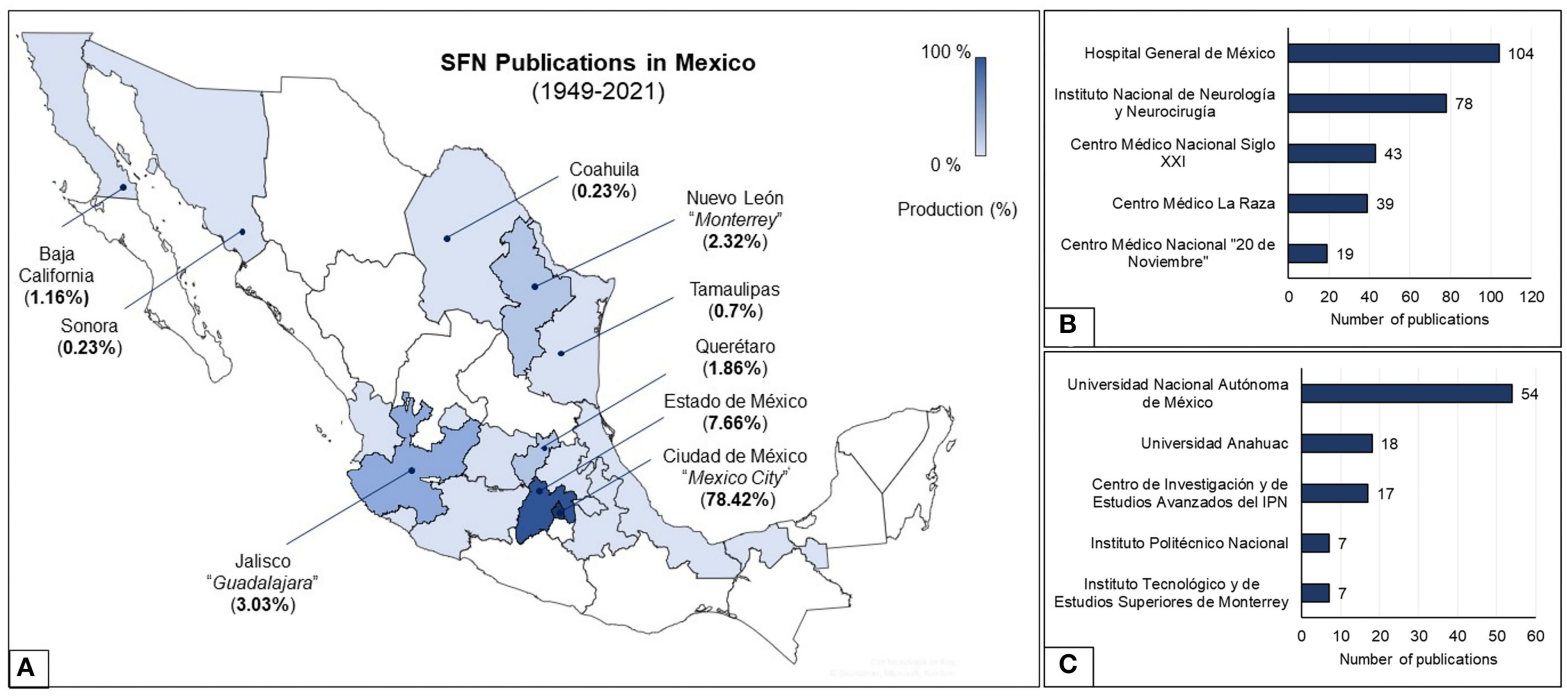
**(A)** Scientific production map of Mexico. **(B)** Most productive neurosurgery departments. **(C)** Most productive universities and research centers.

Scientific production in Mexico is geographically skewed: Mexico City (78%), Guadalajara (3%), and Monterrey (2%) (shown in Figure 4A). Overseas collaboration is basically with the US (34, 9.1%), Canada (12, 3.2%), Germany (8, 2.1%), and Brazil (7, 1.9%). The network was focused on North America, highlighting those peripheral states located on the border (Baja California, Sonora, Coahuila, and Tamaulipas), which have a higher production. The top-producer department was at the HGM (28% of total historical production) (shown in Figure 4B), and the top university was the Universidad Nacional Autónoma de México (UNAM) (14.5%) (shown in Figure 4C). Table 2 illustrates the areas of interest of the most important departments, showing that not all centers conduct research on all topics, and some have a predilection for some areas in particular. An often-neglected bibliometric indicator is the rate of uncited publications. The L0 index is the proportion of uncited items to the total number of papers (8–10). With 102 uncited items from 371, the L0 index is 27.5%. Lower L0 indexes are related to areas with a higher proportion of cited items, and thus more “popular” in terms of citations: spinal cord stimulation (6.9%), history (16.7%), behavior (psychosurgery) (21.5%), epilepsy (25.2%), movement disorders (28.9%), pain (35.6%), neuromodulation (37.5%), and stereotactic biopsies (53.8%).

**Table 2:** Neurosurgery Department's production by subject of research.

**Institution**	**Epilepsy**	*Movement Disorders	**^†^Pain**	**Behavior**	**Spinal Cord stimulation**	**^‡^Neuromodulation**	**Stereotactic biopsies**	**History**	**Total**
HGM	44 (11.85%)	22 (5.92%)	8 (2.15%)	21 (5.66%)	2 (0.53%)	5 (1.34%)	1 (0.26%)	1 (0.26%)	104 (28.03%)
INNN	43 (11.59%)	3 (0.80%)	20 (5.39%)	3 (0.80%)	2 (0.53%)	2 (0.53%)	3 (0.80%)	2 (0.53%)	78 (21.02%)
CMN	14 (3.77%)	4 (1.07%)	2 (0.53%)	4 (1.07%)	9 (2.42%)	2 (0.53%)	0 (0%)	1 (0.26%)	43 (11.59%)
CMLR	2 (0.53%)	16 (4.31%)	4 (1.07%)	0 (0%)	16 (4.31%)	1 (0.26%)	0 (0%)	0 (0%)	39 (10.51%)
CM20	2 (0.53%)	0 (0%)	8 (2.15%)	5 (1.34%)	0 (0%)	2 (0.53%)	1 (0.26%)	1 (0.26%)	19 (5.12%)

HGM, Hospital General de México; INNN, Instituto Nacional de Neurología y Neurocirugía; CMN, Centro Médico Nacional Siglo XXI; CMLR, Centro Médico La Raza; CM20, Centro Médico Nacional “20 de Noviembre”; ^*^ Involve spasticity.^†^Involve trigeminal neuralgia, hemifacial spasms, and peripheral nerve surgical management. ^‡^ Involve different neurological disorders (epilepsy, movement disorders, and pain). Neuromodulation is used routinely for all other categories. However, was considered as a separate section because in Mexico neuromodulation with deep brain stimulation is not a conventional therapy due to the socioeconomic context of the country.

## Discussion

**Figure 5:**
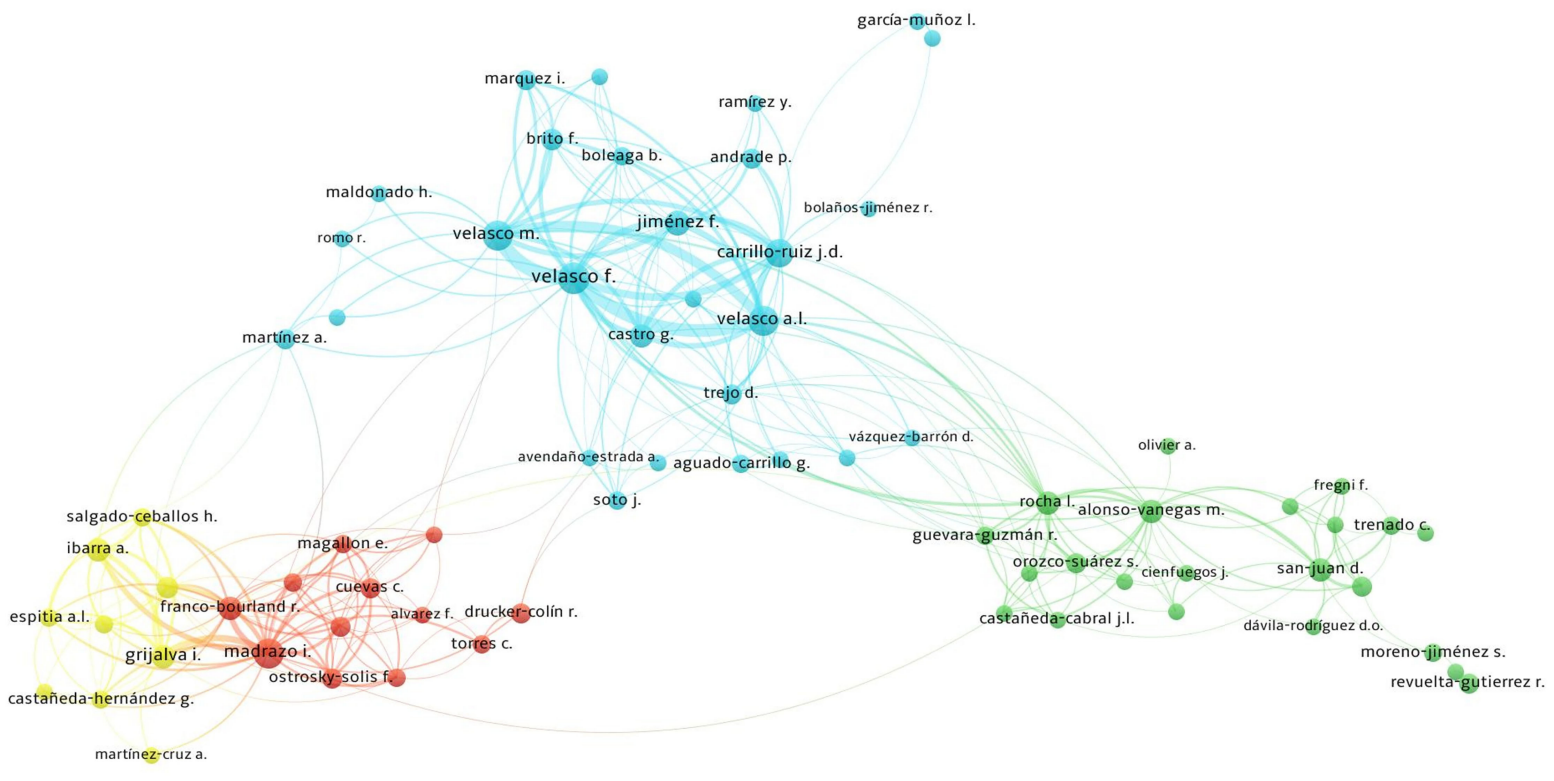
Bibliometric network made in VOSviewer version 1.6.16. The network analysis shows the relationship between the different authors belonging to the most productive institutions in the country, highlighting those authors/groups that presented more collaborations. Institutions are represented by different colors: Hospital General de México (*blue*), Instituto Nacional de Neurología y Neurocirugía (*green*), Centro Médico Nacional Siglo XXI (*red*), and Centro Médico Nacional “La Raza” (*yellow*).

The most influential authors are shown in Table 3, with Francisco Velasco as the most productive neurosurgeon (24.7% of national authorship) and Marcos Velasco (a neurophysiologist) as the most productive non-neurosurgical author (17.8% of national authorship). Francisco Velasco, the most productive neurosurgeon, started working at the neurosurgery department at the HGM in 1972 after completing his training at Johns Hopkins Hospital in Baltimore and in Notre Dame Hospital in Montréal. He founded the Unit of Stereotactic and Functional Neurosurgery at the HGM, training since then prominent and promising functional neurosurgeons [Machado-Salas JP, Jiménez F, Brito F, Carrillo-Ruiz JD, Castro G, Soto J, Navarro-Olvera JL, Aguado-Carrillo G, and Beltran JQ, among others (more than 100 neurosurgeons trained by Francisco Velasco)]. Francisco Velasco's group in the 1990's proposed the centromedian thalamic nuclei and cerebellar cortex as targets for the treatment of patients with intractable generalized tonic-seizures (16–21). In the 2000's, they described the hippocampus as the target for the treatment of refractory complex partial seizures. In addition, they used motor cortex stimulation for patients with epilepsy foci in the primary or supplementary motor areas (22–24). Furthermore, they studied and proposed prelemniscal radiations (in the posterior subthalamic region) as a target for the treatment of motor symptoms of Parkinson's disease (PD) (25–28). Additionally, they described the inferior thalamic peduncle as a punctual target to improve major depression disorder and obsessive-compulsive disorder (29–31), and reported that the bilateral anterior capsulotomy combined with cingulotomy might reduce medically intractable aggressive behavior in specific cases (32). In recognition of his contributions, Dr. Francisco Velasco-Campos was awarded the World Federation of Neurosurgical Societies Medal of Honor in 2015, the FLANC Medal of Honor in 2016, and the Spiegel-Wycis Award in 2017 (6). Other relevant authors shown in Table 3 have focused on other areas, including Madrazo in spinal cord injury, PD, and neurocysticercosis. His top-cited papers deal with adrenal medulla transplantation for intractable PD (33–35), with Alonso-Vanegas making advances on translational research in epilepsy (36), and Revuelta describing the effectiveness of surgical decompression for trigeminal neuralgia (37–39). Figure 5 demonstrates the network analysis between the different authors mentioned previously, showing the collaborations between the most productive groups in the country.

**Table 3:** Leading authors in stereotactic and functional neurosurgery (SFN) in Mexico.

**^†^Leading Neurosurgeons**.	
Authors	^*^Number of publications
Velasco F.	92
Madrazo I.	59
Jimenez F.	52
Carrillo-Ruiz J.D.	46
Alonso-Vanegas M.	34
Revuelta R.	19
Castro G.	17
Brito F.	12
Leading non-neurosurgeon's authors with production in the field of functional neurosurgery.	
Authors	^*^Number of publications
Velasco M.	66
Velasco A.L.	58
Franco-Bourland R	35
Rocha L.	19
Ostrosky-Solis F.	17
^‡^Other relevant authors (Neurosurgeons)	
Aguado-Carrillo G	Moreno-Jiménez S
Arellano-Reynoso A	Navarro-Olvera JL
García-Muñoz L	Pérez de la Torre R
Hernández-Salazar M	Sánchez-Bonilla B
Beltrán JQ	Sandoval-Balanzar MA
Machado-Salas JP	Soto J
Mercado-Pimentel R	
Pioneering authors in stereotactic and functional neurosurgery in Mexico.	
Álvarez-Loyo Jorge	
Escobedo F	
Fernández Dario	
García-Sancho María Cristina	
Velasco-Suarez Manuel	

^*^ *Represent the articles that meet the inclusion criteria for the analysis*.

† *More than ten publications that meet the inclusion criteria for the analysis*.

‡ *Less than 10 publications that meet the inclusion criteria for the analysis*.

One of our limitations was the results of the first search that yielded a total of 225 hits. We noticed that some publications of the most productive neurosurgeons (more than 10 publications during this search) were absent from this list (keywords strategy), probably due to indexation using different synonyms. By selecting these authors and through a search strategy by author, and selecting exclusively the documents pertaining to the field of SFN, we added another 146 documents found in the Scopus database, but not found during the first round. Another limitation was regarding the parameters used to analyze productivity. Number of publications neglects the contribution of the author and the quality of the journal; citations in a negative context or self-citation are also counted. This can be partially controlled by using parameters like the H-index or other alternatives (m-quotient, hc-index, e-index, and g-index) (40). Classically, bibliometrics has focused on the “most cited” elements. However, it was considered important to evaluate the number of articles without citations, using an index proposed by the authors (L0 index), which is obtained by calculating the percentage of articles not cited over the total number of published articles, a bibliometric parameter that can reveal fields that have not gained acceptance. Regarding the bibliometric parameters for journals, this study focused on IF only, as it is less prone to fluctuations or manipulation, while other stricter alternatives are recommended (Eigenfactor, SCImago Journal Rank, Source Normalized Impact per Paper, and Article Influence Score). We all know that IF is limited by its susceptibility to manipulation by editorial policies. Yuen (41) analyzed the correlation between various bibliometric parameters (such as IF, Eigenfactor, AIS, and SJR) in Neurosurgical Journals, showing that they are statistically significantly correlated with one another, and it is feasible to use them as comparable parameters in neurosurgery. For the sake of simplicity, we limited ourselves to standard parameters (number of publications, number of citations, and IF) to enable comparison in further studies (42). Concerning language, the citation ratio in English is 10-fold compared to other languages, which is remarkable considering that the authors and the subject are quite similar, and this adds to the evidence that language of publication is paramount in the dissemination and impact of medical research. Relative to the selected source, Scopus was chosen not only because of citation tracking but also because many Mexican journals are not indexed in the Journal Citation Reports (PubMed/Embase) but are included in Scopus. However, it cannot obtain precise information on the number of citations from 1996 and after, and publications before 1966 are difficult to access. On the other hand, we acknowledge that other publications indexed elsewhere are not included and this limitation may underestimate the importance of Mexican research in this field (43). In addition, the impact of publications may be biased by the practice of self-citation, so in future studies, it might prove more suitable to include an evaluation tool to measure how self-citations increase the impact of a publication.

The main topic of research was epilepsy (31%), followed by movement disorders (22.4%). An epidemiological study in a Latin American population shows a prevalence of epilepsy per 1,000 inhabitants of 10.3 [95% confidence interval (CI): 8.5–13.0], and in PD of 4.7 (95% CI: 2.2–8.9) (44). Although not all neurological disorders are good candidates for neurosurgery, the prevalence of neurological disorders in a Latin American country allows us to estimate the prevalence in Mexico. Our study shows that in fact, the fields of study of SFN in Mexico matches closely with the needs of our population. However, less than 20% of the production focuses on the study of behavioral disorders (11.3%) or pain (15.9%), despite being frequent conditions, so these fields require more attention.

This study showed that publications in English and in high-impact journals show a greater impact (citations). However, there are other factors to consider for impact and productivity. In the field of neurosurgical research, a recent article analyzed approximately 4,000 published articles to reveal factors associated with a greater number of c/p. Documents with a class I level of evidence, collaborations with multiple institutions, IF of the journal, and an increase of 2–4% for each additional contributing author were the factors associated with an increase in the number of c/p according to Oravec et al. (45). Articles produced in international collaboration have a greater impact, with a positive correlation between the number of citations and the number of countries contributing to the publication (46). In our study, we found that publications in English and the publications in journals with an IF > 1 display a greater number of c/p, and stress the relevance of writing skills in English and production in high impact journals (47). Other factors associated with a higher citation rate are academic rank and physician salaries. A positive relationship has been established between the H-index and the academic rank, as well as between the salary granted to physicians and scientific productivity (48, 49). Jamjoom and Jamjoom (50) reported the 50 most important countries in clinical research in neurology according to the number of published documents, establishing Mexico as the 28th country, with a total of 2,602 documents, second only to Brazil in Latin America. Brazil is number 12, with a total of 11,396 items. One of the most significant factors for scientific productivity was the number of universities ranked in the Top 500, and the number of journals indexed in the JCR, where Mexico had just one ranked university and three journals, considering that the gross domestic product (economic aspect) does not have such a strong relationship with productivity (50).

The only report that analyzed the SFN productivity and impact in a global manner was published by Lipsman and Lozano (15), who carried out a bibliometric analysis of the 100 most cited works in SFN. Key findings are data on recent trends in our specialty in terms of citations per year, including the popular topics with a recent focus being brain-machine interface technology, deep brain stimulation for depression/PD disease, and temporal lobectomy for epilepsy. Conversely, relative to the study of SFN fields, movement disorders correspond to 31% of the articles with the greatest impact and are the largest proportion of all categories. In Mexico, the global trends of impact on the SFN are not the same, showing that the object of study with the higher impact was epilepsy, corresponding to 31% of the historical production in Mexico and 33.1 citations per article. In contrast with Lipsman and Lozano's (15) article, the merit of this study was to be the first analyzing the scientific production from the field of SFN in a single country, trying to find those factors specifically associated with productivity and the impact of that country that could benefit the research output. The reproduction of this type of analysis in multiple countries can reveal points of comparison and factors associated with the impact of scientific production at a national level, which will be useful in revealing gaps in research and therefore areas that merit further development. Bibliometric analysis has been useful in other areas of knowledge outside neurosurgery (51, 52). Bibliometric analysis is the map of science, as it precisely maps the areas of interest (cities), most popular published topics (highways), highly cited-items (skyscrapers), over-explored fields (traffic jams), neglected subjects (deserts), uncited items (wasteland), and sleeping beauties (hidden pirate treasures).

## Conclusions

With steady growth for half a century, Mexico has also seen a steep increase in the productivity on SFN literature, addressing the most prevalent issues of epilepsy and motor disorders in the country. However, it is necessary to report studies with a higher level of evidence, as well as to decentralize the research by collaborating with Mexican institutions outside Mexico City. On the other hand, it is relevant to promote scientific production in English and in high-impact indexed journals to increase the impact of our scientific output. We would like to call on our colleagues to reproduce this study in their countries, in order to determine the factors associated with the impact and productivity of SFN research.

## Data Availability Statement

The raw data supporting the conclusions of this article will be made available by the authors, without undue reservation.

## Author Contributions

JC-R, AA-S, and ÁL: conceptualization and methodology. JC-R, AA-S, JN-O, JB, and ÁL: software, data curation, writing, original draft preparation, and visualization. BB and GG-G: investigation. ÁL: supervision. JN-O and JB: software and validation. JC-R and AA-S: writing–reviewing and editing. All authors contributed to the article and approved the submitted version.

## Conflict of Interest

The authors declare that the research was conducted in the absence of any commercial or financial relationships that could be construed as a potential conflict of interest.

## Publisher's Note

All claims expressed in this article are solely those of the authors and do not necessarily represent those of their affiliated organizations, or those of the publisher, the editors and the reviewers. Any product that may be evaluated in this article, or claim that may be made by its manufacturer, is not guaranteed or endorsed by the publisher.

## Abbreviations

CMLR: Centro Médico Nacional “La Raza”
c/p: Citation per publication
DBS: Deep brain stimulation
HGM: Hospital General de México
IF: Impact factor
INCMNSZ: Instituto Nacional de Ciencias Médicas y Nutrición Salvador Zubirán
INNN: Instituto Nacional de Neurología y Neurocirugía
PD: Parkinson's disease
SFN: Stereotactic and Functional Neurosurgery
TMS: Transcranial magnetic stimulation
UNAM: Universidad Nacional Autónoma de México.
